# The specific phagocytosis regulators could predict recurrence and therapeutic effect in thyroid cancer: A study based on bioinformatics analysis

**DOI:** 10.1097/MD.0000000000033290

**Published:** 2023-03-17

**Authors:** Changran Hou, Mengmeng Wu, Haojie Zhang, Zhenlin Yang

**Affiliations:** a Department of Thyroid and Breast Surgery, Binzhou Medical University Hospital, Binzhou, Shandong, P.R. China; b Binzhou Medical University, Yantai, Shandong, P.R. China.

**Keywords:** CRISPR-cas9, phagocytosis regulators, therapeutic effect, thyroid cancer, tumor recurrence

## Abstract

Thyroid cancer (TC) is one of the growing cancers and is prone to recurrence. Meanwhile, in immunotherapy, antibody-dependent cellular phagocytosis (ADCP) phagocytosis related regulators (PRs) play an important role. This study aims to investigate the prognostic value of specific PRs in TC.

**Methods:**

The purpose of this study was to identify specific PRs in TC patients by retrieving RNA-seq and Clustered Regularly Interspaced Short Palindromic Repeats-cas9 data and an algorithm based on LASSO was used to construct the PRs-signature. Subsequently, prognosis value of PRs-signature for recurrence-free survival (RFS) was explored through various statistical analysis, including Cox regression analysis, Kaplan–Meier analysis, and receiver operating characteristic curve. Additionally, an analysis of immune cell content by risk group was conducted using CIBERSORT, single sample gene set enrichment analysis and MCP-counter algorithms, with a particular focus on the correlation between macrophages and specific PRs.

**Results:**

We identified 36 specific PRs, and a PRs-signature was constructed using 5-prognostic PRs (CAPN6, MUC21, PRDM1, SEL1L3, and CPQ). Receiver operating characteristic analysis showed that predictive power of PRs-signature was decent, and the PRs risk score as an independent prognostic factor was found to be correlated with RFS showed by multivariate cox regression analysis. Meanwhile, a lower RFS was observed in the high-risk group than in the low-risk group. The results of the 3 algorithms suggested that our PRs-signature may have certain significance for macrophage content and ADCP. Interestingly, the low-risk group had higher levels of mRNA expression than the high-risk group at PDCD1, CTLA4, and pro-inflammatory factors from macrophage.

**Conclusion:**

For the purpose of prognostic management, this study developed a prediction model. And the cross-talk between certain PRs and TC patients was revealed in this study. Besides, the PRs-signature can predict the immunotherapy response, macrophage content, and ADCP status. TC patients will benefit from these developments by gaining insight into novel therapeutic strategies.

## 1. Introduction

Thyroid cancer (TC) is one of the most rapidly increasing cancers.^[1]^ Luckily, TC has an excellent prognosis, with a 5-year survival rate of nearly 97% based on current follow up reports.^[2]^ Although TC has a low mortality rate, it has a 20% to 30% recurrence, which is higher in individuals with variant types.^[3]^ In order for TC patients to obtain the most optimal dosage regimen, it is critical to appropriately quantify the risk of recurrence. Although previous research has explored encouraging findings,^[4]^ the recurrence predictor still has to be thoroughly investigated in TC.

Several biological functions are associated with phagocytosis. These include clearing apoptotic cells, regenerating cells, monitoring tumors, and removing cellular debris after damage.^[5]^ Simultaneously, deficiencies in phagocytosis can result in autoimmunity and developmental disorders.^[6]^ In addition, to engulf various types of particles, phagocytes use diverse surface receptors and signaling cascades.^[7]^ Among the important aspects of monoclonal antibody therapies targeting tumor antigens is that they trigger macrophage phagocytosis of cancer cells, which is an important part of cancer cell elimination.^[8]^ Thus, tumor immunotherapy depends on identifying antibody-dependent cellular phagocytosis (ADCP)-related regulators. Luckily, the development of the Clustered Regularly Interspaced Short Palindromic Repeats (CRISPR)/Cas9 system has enabled dramatically improved genome-scale knockout screens with high exactness in mammalian cells.^[9]^ Thus, ADCP phagocytosis related regulators (PRs) have been identified at a large scale using this method by the researchers.^[8,9]^ Nevertheless, a thorough study of the prognostic relationship between PRs and thyroid cancer is lacking.

Thus, the purpose of this study was to advance a new prognostic signature based on above PRs for predicting recurrence-free survival in patients with TC. Additionally, further validation of the tumor immune microenvironment and response to ICI therapy was conducted. Particularly, a study was conducted to investigate the association between specific PRs and macrophages in thyroid carcinoma tissues, as well as the feasibility of assessing ADCP status via PRs.

## 2. Materials and Methods

### 2.1. Datasets and data preprocessing

A total of 502 TC samples and 58 normal samples in total were included in this study. RNA-seq and clinical data were obtained from TCGA databases (FPKM-level3).^[10]^ The TCGA-TC cohort was randomly divided into 6:4 and represented as training set and testing set. Concurrently, IMvigor210,^[11]^ the ATE zolizumab (anti-PD-L1 antibody) cohort to treat uroepithelial carcinoma, was extracted to assess the predictive value of PRs-signature as an immunotherapy predictor. Additionally, CRISPR was used to identify 730 genes that regulate cancer cell phagocytosis in the *Nature journal*.^[9]^

### 2.2. PRS risk score identification and validation

According to RNA-seq data obtained from TCGA database, the differential expression PRs list (adj. *P* value < .05, |log FC| > 1), as specific PRs in TC.^[12]^ Using the single sample gene set enrichment analysis (ssGSEA) algorithm^[13]^ and Spearman analysis at the same time, we calculated macrophage content in TC tissues and explored the interrelation between specific PRs and macrophages. Subsequently, through univariate Cox regression and Kaplan–Meier survival analysis, we screened out prognostic PRs from specific PRs (*P* value < .1). In training set, LASSO regression analysis^[14]^ was conducted for the above genes. The risk score was calculated as follows: $\underset{i=1}{\overset{n}{\sum }}\;Coef_{i}*x_{i}$. It is worth noting that the coefficient of multivariate Cox regression is called Coef, the expression value of each adopted gene is x. Area under the curve and receiver operating characteristic curves were used to assess the PRs-signature’s ability to predict survival. Based on the median value of the risk score for each patient we calculated, we were able to define “high-risk” and “low-risk” groups. Various groups were analyzed using Kaplan–Meier methods to determine differences in recurrence-free survival (RFS).^[15]^

### 2.3. Comprehensive analysis of immune and molecular characteristics

To examine the biological processes related to specific PRs, KEGG and GO enrichment analyses were performed.^[16]^ We investigated the immune characteristics of the TC samples by importing expression data into CIBERSORT to evaluate the proportion of 22 immune cells,^[17]^ and to further validate the algorithm, we used the ssGSEA algorithm and MCP-counter^[18]^ algorithm. Eventually, a comparison of immune checkpoint and pro-inflammatory factor mRNA expression levels were conducted between groups. Additionally, 2 sub-consensuses were formed among TC patients by NMF package^[19]^ in R software based on the specific PRs expression levels. The number of sub-consensuses was determined by the number of ranks.

### 2.4. Comparison of prediction efficiency

To explore the prognostic value of PRs in patients after immunotherapy, survival analysis was performed in IMvigor210 cohort. As a means of demonstrating PRs powerful prognostic value, we compared them to other signatures from a variety of references. Han et al^[20]^ recognized a soluble carrier family genes signature for patients with TC, SFXN1, SLC12A4, SLC35A1, SLC35E1, and SLCO1C1 included. In addition, Wen et al^[21]^ constructed a lipid metabolism-related genes signature for recurrence possibility of TC patients, including PDZK1IP1, TMC3, LRP2 and KCNJ13. Eventually, models were compared using C-index to determine which had the best prognostic ability.

### 2.5. Quantitative real-time PCR

We collected 10 tumor samples as well as normal samples from tumor resected TC patients. The normal tissue was 2 to 3 cm away from the tumor during sampling during collection. A Medical Ethics Committee approved the collection of all tissue samples from the Thyroid and Breast Surgery Department at Binzhou Medical University Hospital. We detected the expression of CAPN6, MUC21, PRDM1, SEL1L3, and CPQ in thyroid cancer and Normal thyroid tissue by Quantitative real-time PCR. As directed by the manufacturer, total RNA was extracted from fresh tissue with Trizol (B610409-0100, Sangon Biotech). An RNA sample was reverse transcribed into cDNA using the Revert Aid First Strand cDNA Synthesis Kit (Thermo Scientific). We used the following amplification protocol: 5 minutes of denaturation at 95°C followed by 45 cycles, including 95°C for 15 seconds, 56°C for 30 seconds, and 72°C for 20 seconds. Use the ΔΔCt method for the calculation of relative expression levels, the target genes relative expression levels were normalized to Gapdh levels. Following is a list of primer sequences. CAPN6 (forward:5′-GTCCTGCCCTAACCCAAGTC-3; reverse:5′-GCCCCTCTCACTGTCTAGGA-3). MUC21 (forward:5′-TAGCACCTCTGCCAACACTG-3; reverse:5′-GGTCACGCTGGACCCT).

PRDM1 (forward:5′-AACACAGACAAAGTGCTGCC-3; reverse:5′-CAAGGTCGTACCCACACGTT-3). SEL1L3 (forward:5′-CTGCTCCTGCTCTGCTACC-3; reverse:5′-CTTTGTAAGCCACGCTCTGC-3). CPQ (forward:5′-GATGGGGGCAAAGACCTACC-3; reverse:5′-GAAGGCACCAACTCCACCTT-3). GAPDH (forward:5′-AGAAGGCTGGGGCTCATTTG-3; reverse:5′-AGGGGCCATCCACAGTCTTC-3).

### 2.6. Statistical analysis

Quantitative data from 3 independent experiments were analyzed by Student *t* test and results were expressed as mean ± SD. The statistical analyses were conducted using the R software (v.4.0.1) (http://cran.r-project.org/src/base/R-4/R-4.0.1.tar.gz). An overview of statistical methods for handling transcriptome data can be found in the section above. ***, **, * and ns represent *P* < .001, <.01, <.05, and not significant, respectively.

## 3. Results

### 3.1. Macrophage-mediated ADCP may involve specific PRs in TC

**Figure 1.**
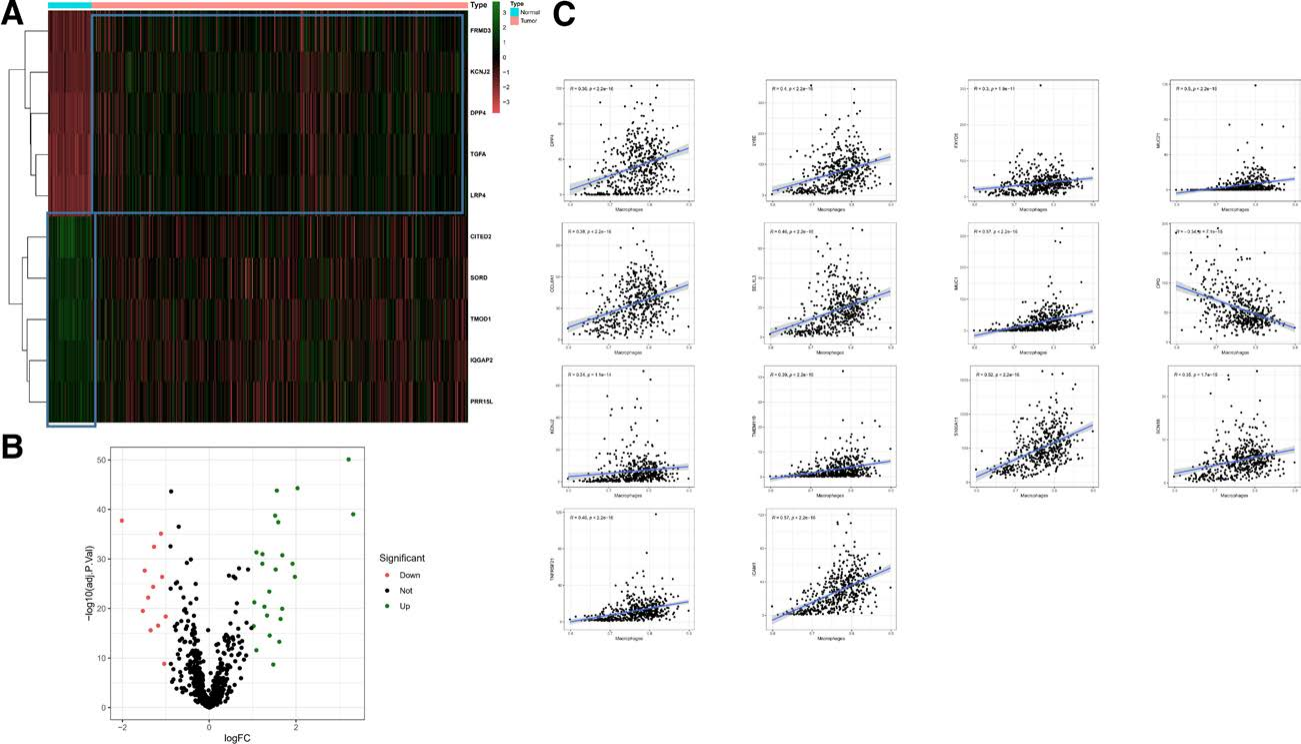
Identification of specific PRs in TC samples. (A) 58 normal samples and 502 TC samples were used for the heatmap to display the top 10 specific PRs. (B) In the volcanic plot, we can see how specific PRs are up regulated and downregulated. (C) A scatter diagram showing 14 specific PRs that correlate with macrophages. PRs = phagocytosis related regulators, TC = thyroid cancer.

CRISPR has been used to identify regulators that block antigen-dependent cell phagocytosis (ADCP), a process by which monoclonal antibodies target tumor antigens trigger macrophages to engulf cancer cells.^[22]^ Thereby, specific PRs were identified using differential expression analysis by analyzing RNA-sequence data of TCGA-TC cohort. Finally, a total of 36 specific PRs were identified in 502 TC tissues and 58 normal samples (Fig. 1A and B). Meanwhile, we used ssGSEA algorithm to estimate the score of macrophages in TCGA-TC tissues, so as to further explore the relationship between specific PRs and macrophages. From 36 specific PRs, 14 PRs were identified to be strongly correlated with macrophage score (*P* < .001, |r|>0.3). It was noteworthy that the strongest negative correlation with macrophages was found with ICAM1 (*R* = 0.57), while only CPQ showed the positive correlation with macrophages in the TC tissues (r = −0.34), as shown in Figure 1C. Taken together, our data suggested that ADCP process in TC tissue may be mediated by the above specific PRs.

### 3.2. Biochemical processes associated with identification of PRs

**Figure 2.**
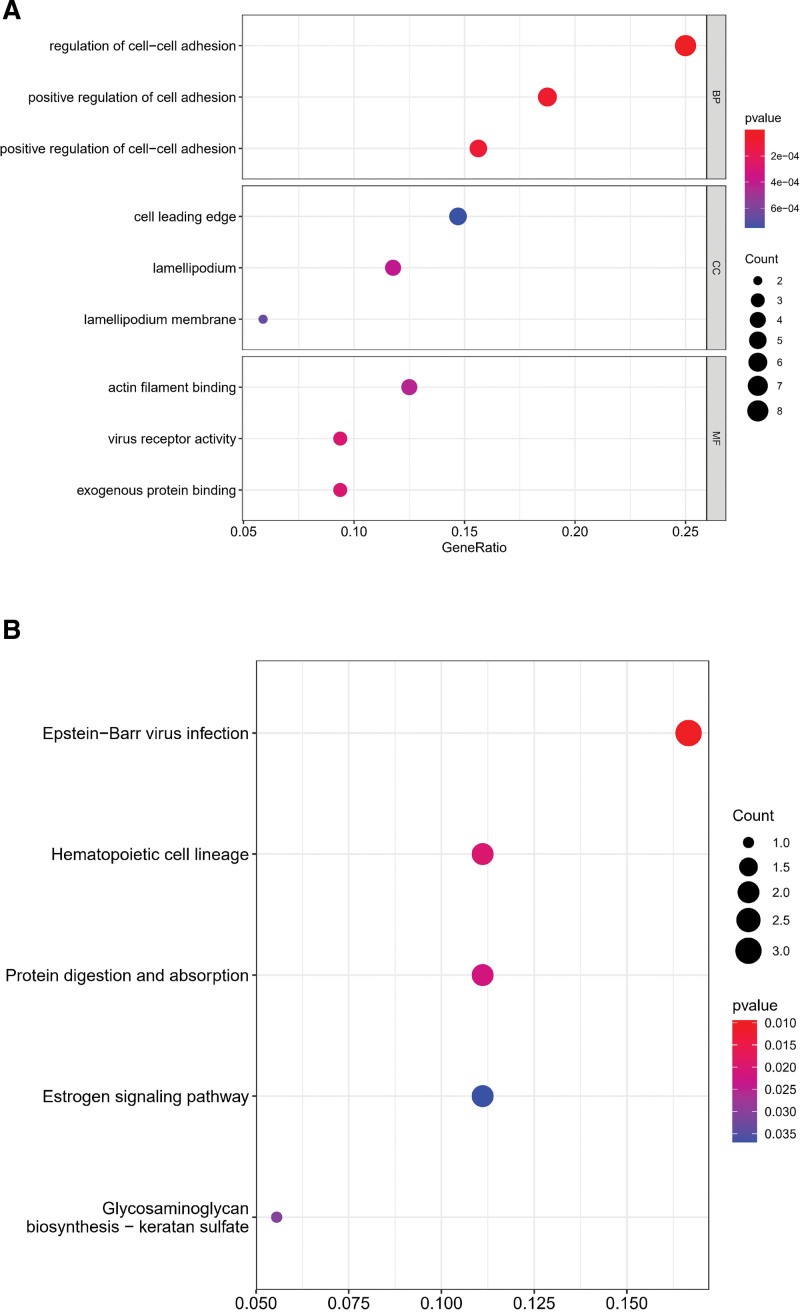
Enrichment analysis. (A) An analysis of 36 specific PRs based on GO enrichment. (B) An analysis of 36 specific PRs based on KEGG enrichment. PRs = phagocytosis related regulators.

In order to elucidate the biological functions of particular PRs, we performed GO and KEGG enrichment analyses. According to GO enrichment analysis, 36 specific PRs were primarily associated with cell-cell adhesion in BP section, cell leading edge, and lamellipodium in CC section, and actin filament biding, and virus receptor activity in MF section (Fig. 2A). Additionally, based on KEGG enrichment analysis, related PRs were enriched related to Epstein-Barr virus infection, hematopoietic cell lineage, protein digestion and absorption, etc. (Fig. 2B).

### 3.3. Specific PRs as potential prognostic indicators of TC

**Figure 3.**
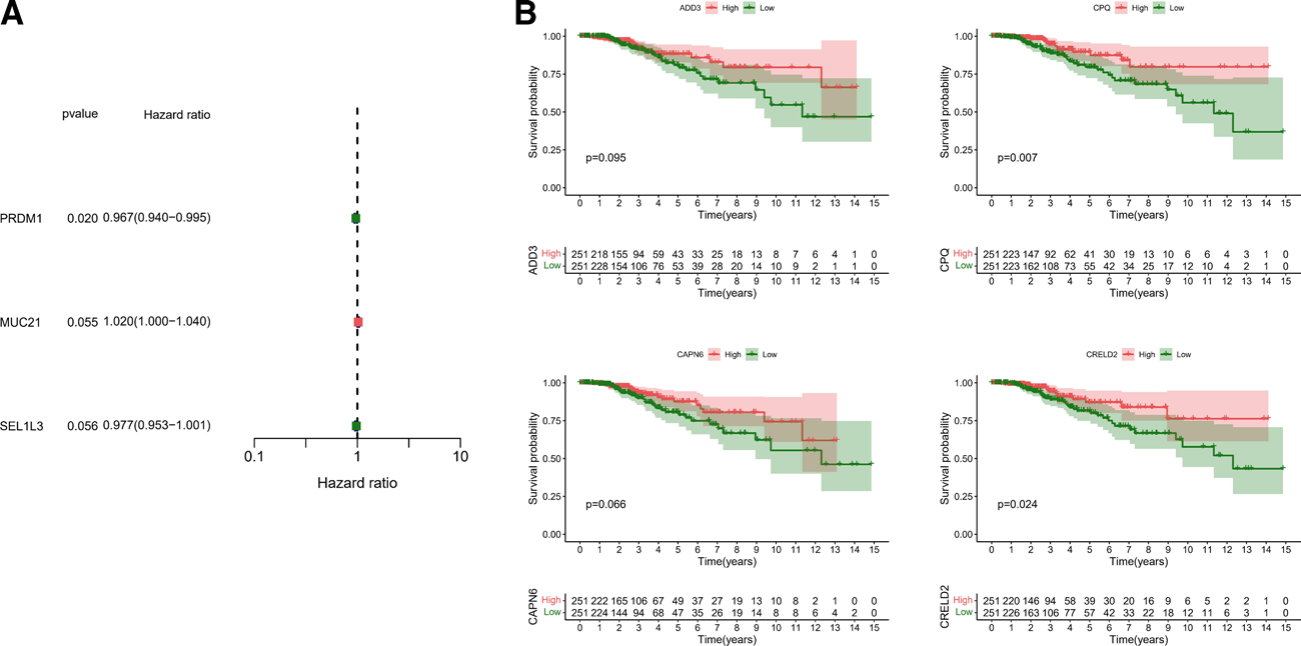
Value of specific PRs as prognostic indicators. (A) forest plot depicted the prognostic specific PRs from a univariate Cox regression, including PRDM1, MUC21, and SEL1L3. (B) 4 prognostic specific PRs were identified by Kaplan–Meier analysis, including ADCD3, CPQ, CAPN6, and CREL02. PRs = phagocytosis related regulators.

Considering the favorable prognosis of TC patients, but some patients are prone to local recurrence. Therefore, RFS was used as the dependent variable in Cox regression analysis and Kaplan–Meier analysis. Firstly, univariate Cox regression analysis (*P* < .1) preliminary screening of specific PRs, and the results showed that 3 PRs were significantly correlated with the RFS of TC patients (Fig. 3A). In addition, Kaplan–Meier analysis and log-rank test (*P* < .1) were again identified in 36 specific PRs, and 4 PRs proved to be RFS indicators (Fig. 3B). Notably, Kaplan–Meier analysis revealed that CPQ was the finest prognostic protective indicator. Taken together, our data suggested that PRDM1, MUC21, SEL1L3, ADD3, CPQ, CAPN6, and CRELD2 were identified as potential prognostic markers.

### 3.4. Quantify the predictive value of PRs on recurrence-free survival in TC patients

**Figure 4.**
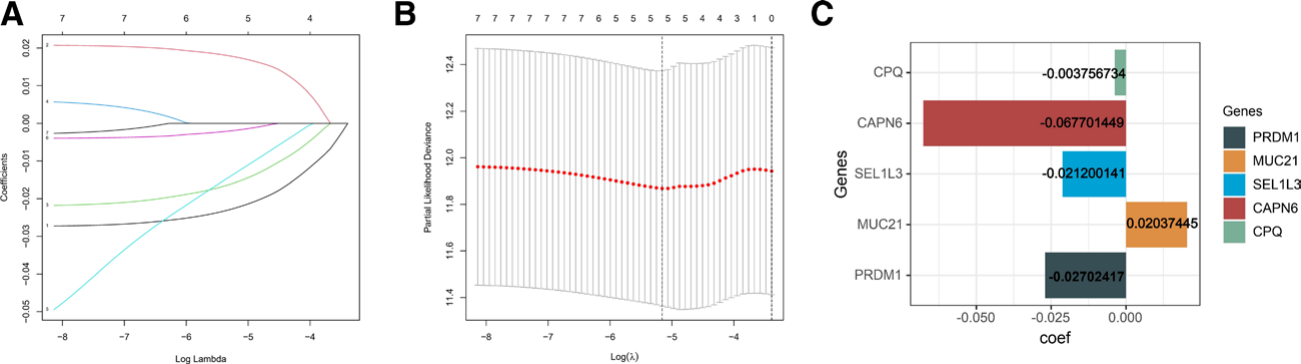
Analyze the predictive value of PRs for recurrence-free survival. (A–B) Laaso regression analysis in RDM1, MUC21, SEL1L3, ADCD3, CPQ, CAPN6, and CREL02. (C) Modeled regression coefficients for every specific PRs. PRs = phagocytosis related regulators.

A lack of phagocytic activity of macrophages and the presence of antiphagocytic factors in cancer cells still hinder targeted therapy. Dysregulation of PRs expression will also negatively impact the survival of TC patients. Therefore, we quantify PRs expression for the prognosis assessment of TC patients to reflect the phagocytosis in tumor tissues. By analyzing Lasso regression in the training cohort using the 7-PRs mentioned above, redundant genes were further removed. (Fig. 4A and B), and the predictive effect performed best when 5-PRs were used. In addition, further Cox regression identified the coefficient of 5-PRs participating in the risk signature (Fig. 4C). Hence, the formula of PR-risk score = (−0.0037 × CPQ expression level) + (−0.0677 × CAPN6 expression level) + (−0.0212 × SEL1L3 expression level) + (0.0203 × MUC21 expression level) + (−0.0270 × PRDM1 expression level).

### 3.5. Validation of clinical applicability of PRs risk Score and nonnegative matrix factorization algorithm for identifying molecular subtypes

**Figure 5.**
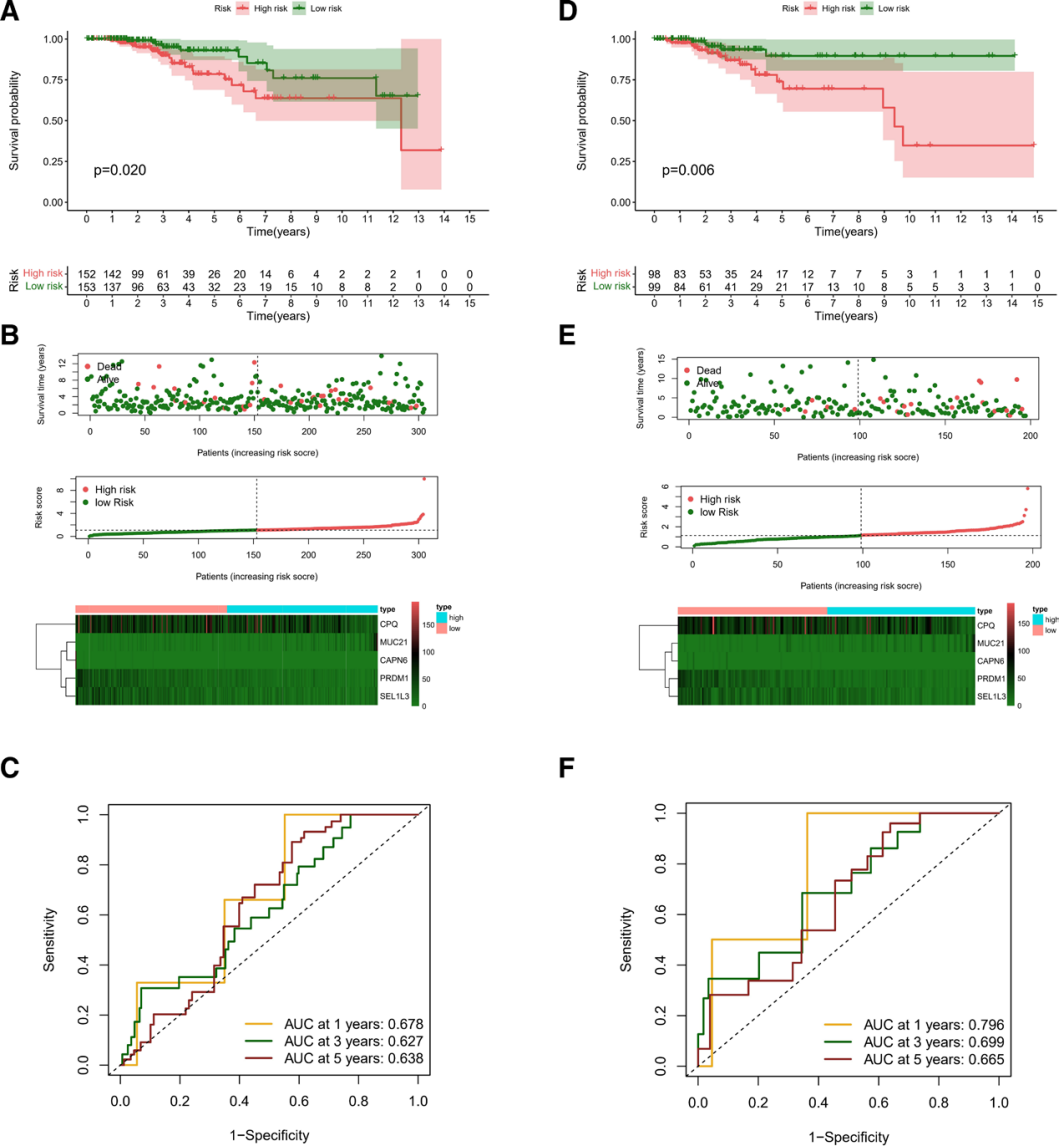
predictive value validation. (A) Cohort modeling with Kaplan–Meier analysis. (B) Distribution of survival in the cohort modeled. (C) Modeling cohorts with 1-, 3-, and 5-year ROC analyses. (D) Cohort validation using Kaplan–Meier analysis. (E) Distribution of survival in the validation cohort. (F) The ROC analysis for the validation cohort at 1, 3, and 5 years. ROC = receiver operating characteristic.

**Figure 6.**
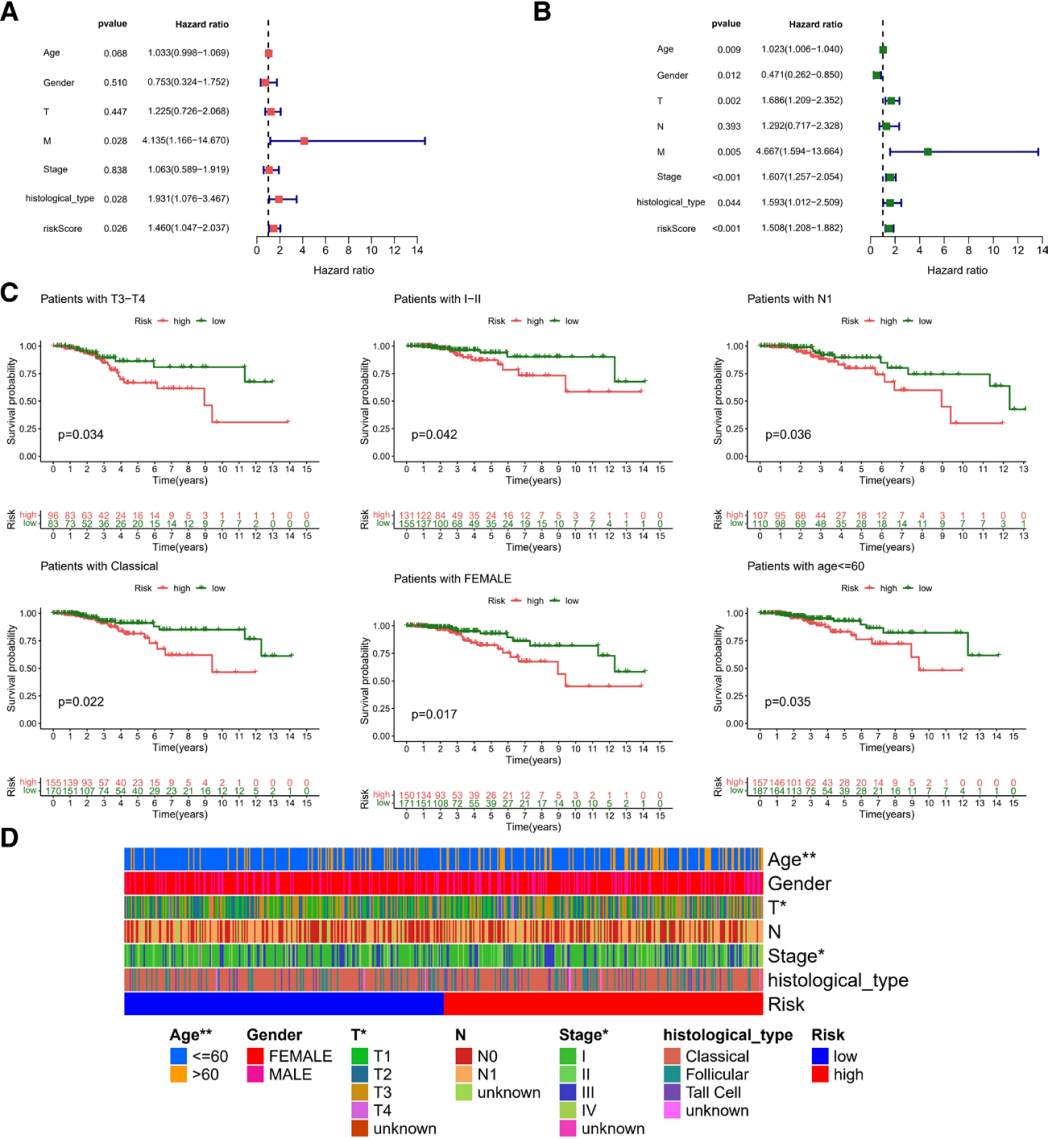
Clinical correlation analysis and independent prognostic value evaluation. (A) Univariate Cox regression in age, gender, T staging, M staging, clinical stages, histological type, and PRs risk score. (B) The analysis of multivariate Cox regression on the clinical features above. (C) Kaplan–Meier analysis in clinical subgroup. (D) Clinical correlation analysis in different PRs risk group. *P < .05, **P < .01, ***P < .001. PRs = phagocytosis related regulators.

**Figure 7.**
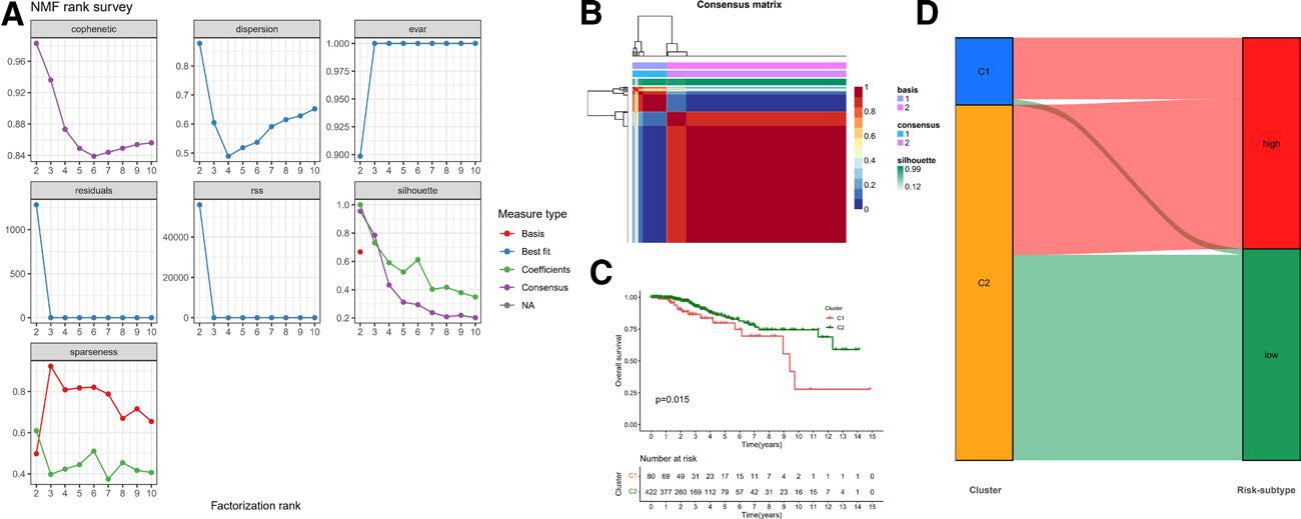
Nonnegative matrix factorization algorithm for identifying molecular subtypes. (A) NMF rank survey list, including cophenetic, dispersion, EVAR, residuals, RSS, silhouette, sparseness. (B) A Heatmap in consensus matrix for identifying 2 cluster. (C) Kaplan–Meier analysis in different molecular cluster. (D) Sankey diagram of molecular subtypes and risk groups.

All patients in the training and testing cohorts were classified as low-risk and high-risk group on the basis of the PR-risk score’s median score. RFS was significantly different between low-risk and high-risk groups based on Kaplan–Meier survival analysis (Fig. 5A and D). Specifically, low-risk group RFS time was longer than high-risk group. Additionally, the distribution of the PR-risk scores also showed that patients with high-risk conditions were more prone recurrence status (Fig. 5B and E). Training cohort receiver operating characteristic analysis showed decent predictive power for risk score (1-year = 0.678, 3-year = 0.627, and 5-year = 0.638), as shown in Figure 5C. Likewise, testing cohorts also demonstrated decent predictive capabilities (1-year = 0.796, 3-year = 0.699, and 5-year = 0.665), as shown in Figure 5F. Meanwhile, throughout all cohorts, the PR-risk score correlated remarkably with the RFS, both in univariate and multivariate analyses of cox regression (Fig. 6A), specifically, it was found that DFS was associated with risks core as an independent risk factor in patients with TC (Fig. 6B). Survival analyses were performed again for clinical subgroups. In *T*3 to *T*4 subgroup, I to II stage subgroup, N1 subgroup, classical pathologic type subgroup, female subgroup, and young subgroup, the PRs risk score can be used as a robust indicator about recurrence (Fig. 6C). Finally, we also explored the distribution of PR-risk scores in different clinical features. Results showed significant statistical differences in commonly used clinical indicators, such as age, *T* staging, and clinical staging (Fig. 6D). Based on the expression of 36 specific PRs in TC mentioned above, molecular subgroups were preliminarily classified by NMF consensus clustering (*C*1, and *C*2), and 2 cluster were divided in TCGA-TC cohort (Fig. 7A and B). Interestingly, different molecular cluster also differ in RFS time similar to risk subgroup (Fig. 7C). The RFS time of the *C*1 subgroup was shorter than *C*2 subgroup. Sankey diagram showed that most of *C*1 subtype belonged to the high-risk group (Fig. 7D).

### 3.6. ADCP status may vary among patients in different risk groups

**Figure 8.**
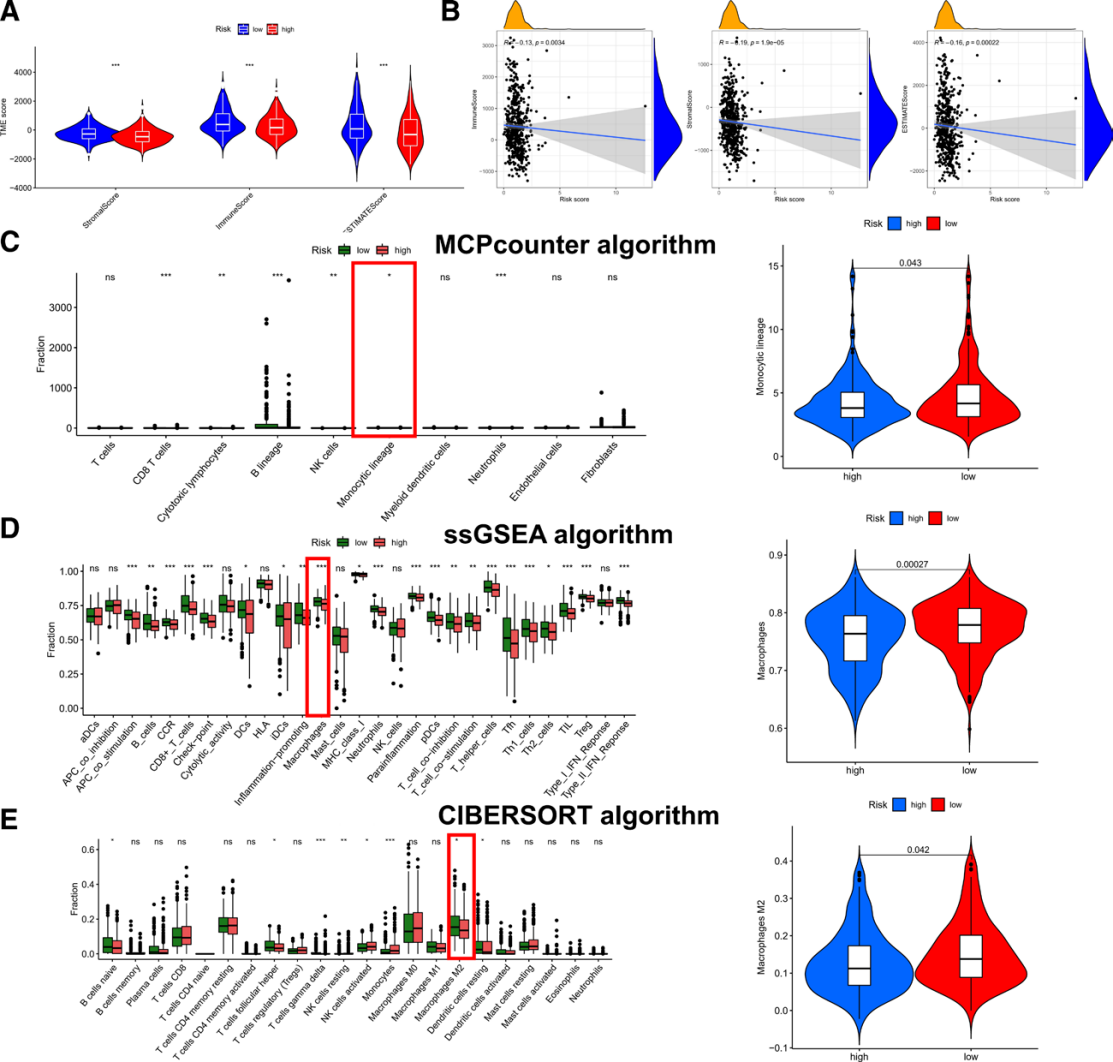
Use MCP-counter, ssGSEA, and CIBERSORT Algorithms for exploring immune status among different PRs risks groups. (A) A comparison of stromal score, immune score, and ESTIMATE score between PRs risk groups. (B) Correlation analysis between PRs risk score and 3 scores. (C) Calculation of immune cell differences using MCP counters. (D) SsGSEA algorithm calculates differences in immune cells. (E) Calculation of immune cell differences using CIBERSORT. *P < .05, **P < .01, ***P < .001. PRs = phagocytosis related regulators, ssGSEA = single sample gene set enrichment analysis.

We evaluated the tumor microenvironment (TME) status in the TC tissues using the ESTIMATE algorithm. Interestingly, there were higher scores on stromal, immune, and ESTIMATE in PRs high-risk group (Fig. 8A). Then, we conducted Spearman correlation analysis on risk score and TME scores, and PRs risk scores and TME scores were positively correlated in the results (Fig. 8B). Considering the important role of macrophages from monocytic lineage in the process of ADCP, we focused on the analysis of macrophages next. Using MCP-counter algorithm, we characterized immune cell types (Fig. 8C). Intriguingly, MCP-counter score shows different distributions of monocytic lineages, and a higher score in the low-risk group. Meanwhile, ssGSEA algorithm was used for an exhaustive analysis of monocytic lineage, and the results showed that macrophages still had a higher abundance in the monocyte lineage in the low-risk group (Fig. 8D). Eventually, a more detailed results of immune cell in CIBERSORT algorithm showed that macrophages M2 had higher expression content in low-risk group (Fig. 8E). Taken together, the results of the 3 algorithms suggested that PRs risk score may have certain significance for macrophage content and ADCP status.

### 3.7. Indicators for estimating immune checkpoint status and pro-inflammatory state are PR-risk scores

**Figure 9.**
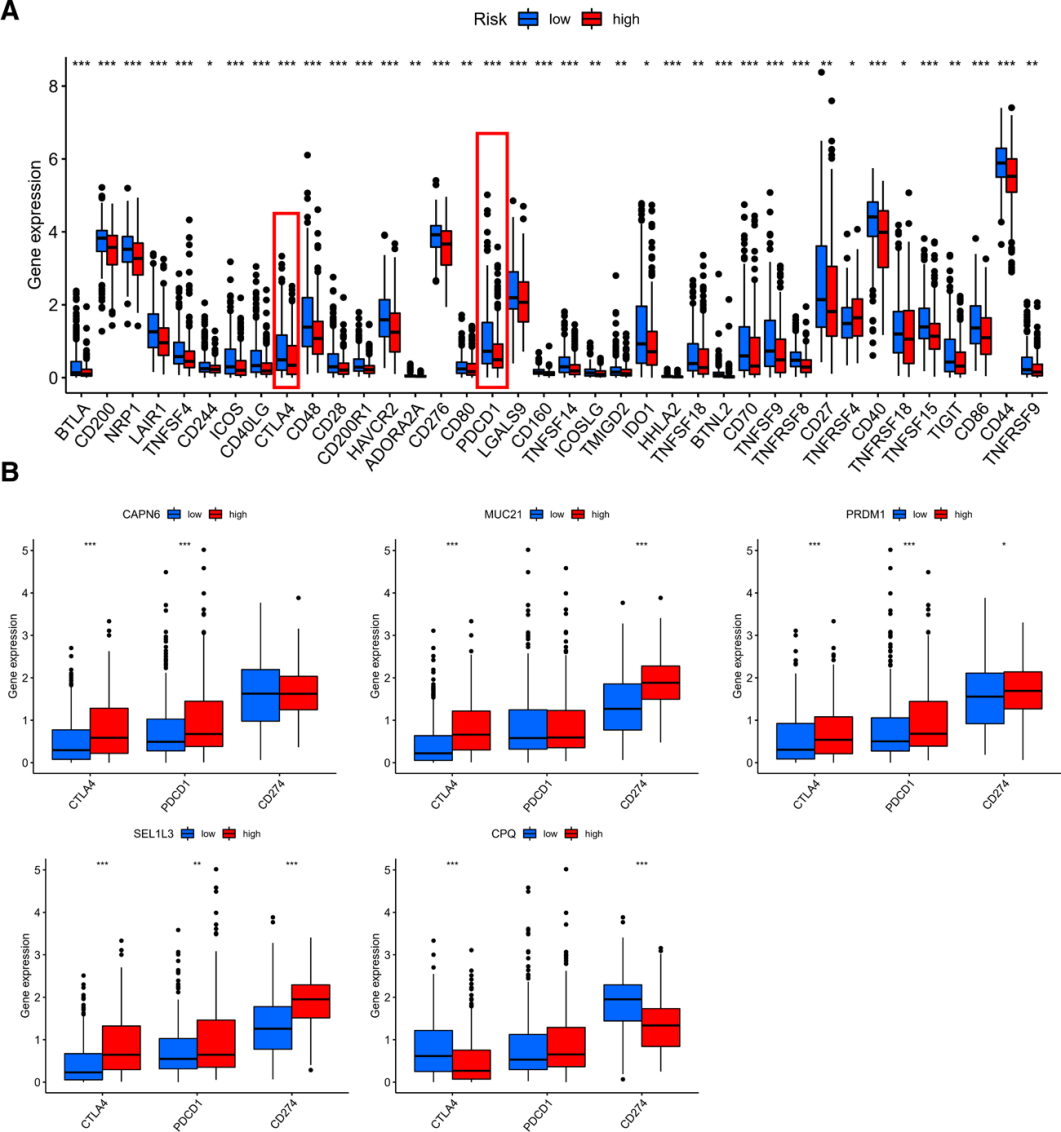
Different PRs risk groups have different potential immune checkpoints. (A) Different risk groups express remarkable immune checkpoints. (B) Difference in CTLA4, PDCD1, and CD274 expression in CAPN6, MUC21, PRDM1, SEL1L3, and CPQ groups. PRs = phagocytosis related regulators.

**Figure 10.**
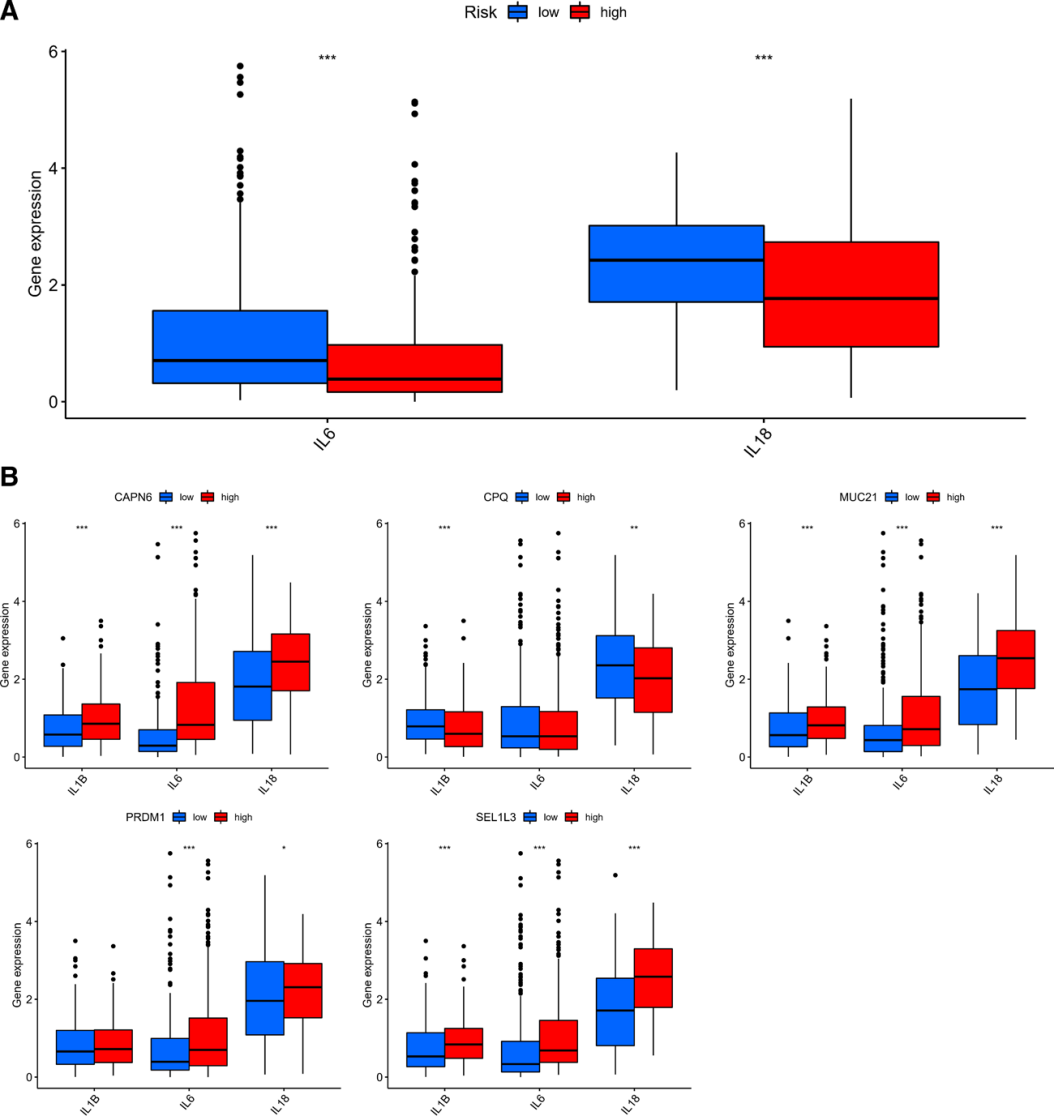
Exploring difference in major ILs from macrophages. (A) Significant IL (IL-16, and IL-18) Different risk groups have different expressions. (B) Difference of IL-16, IL-18, and IL1B expression in CAPN6, MUC21, PRDM1, SEL1L3, and CPQ groups. *P < .05, **P < .01, ***P < .001. IL-18 = interleukin-18.

The purpose of this study is to investigate whether immune checkpoints are associated with risk scores, the immune checkpoint expression of different risk groups were calculated (Fig. 9A). Interestingly, low-risk groups had higher PDCD1 and CTLA4 mRNA expression than high-risk groups, which was currently commonly used for immune checkpoints in cancers. In addition, the expression of PD-1, CD274, and CTLA4 were significantly different between the high-and low-groups in 5 selected PRs (Fig. 9B). A number of studies have demonstrated that chronic inflammation contributes to immune cell infiltration and major pro-inflammatory factors, interleukin-1 α, interleukin-1 β, interleukin-6, and interleukin-18 ^[23]^ included. For this reason, we explored the associations of 3 major ILs from macrophages with PRs risk score. Low-risk participants expressed higher levels of interleukin-6 and interleukin-18 than those at high-risk (Fig. 10A). In addition, as shown in Figure 10B, the 3 pro-inflammatory factors showed difference in high-and low-expression groups.

### 3.8. Comparison of prediction efficiency of different risk signatures

**Figure 11.**
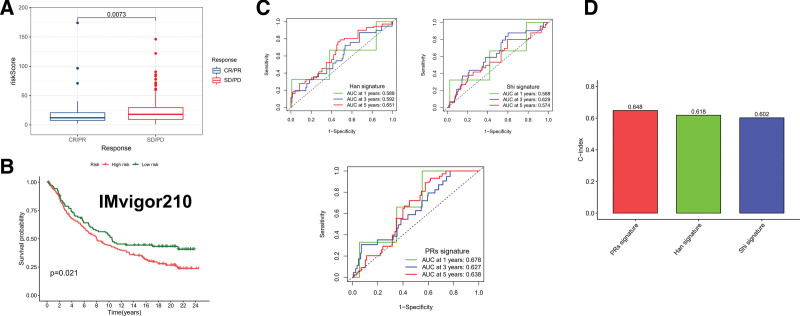
The assessment of immunotherapy and the comparison of different risk models. (A) Differences in risk scores between groups responding to different treatments. (B) Kaplan–Meier survival analysis of anti-PD-L1 cohort (IMvigor210). (C) An analysis of the ROC curves of different risk signatures in the TCGA-TC cohort. (D) C-index comparison in different DFS risk signatures. ROC = receiver operating characteristic, TC = thyroid cancer.

As part of the study, we evaluated the prognostic significance of PRs risk scores in PD-L1-treated patients. It can be found that in CR/PR and SD/PD cohorts, there are significant differences in risk score (Fig. 11A). Meanwhile, there was a better OS for the low-risk group than for the high-risk group, as shown by the results (Fig. 11B). Finally, the prognostic value of PRs risk score was compared with other risk signatures (Fig. 11C). C-index results showed that the PRs risk score performed best in terms of prediction (Fig. 11D). Nevertheless, there is no doubt that the stratification of patients at risk for TC can also be influenced by other risk signatures.

### 3.9. Validation of expression levels about 5-PRs

**Figure 12.**
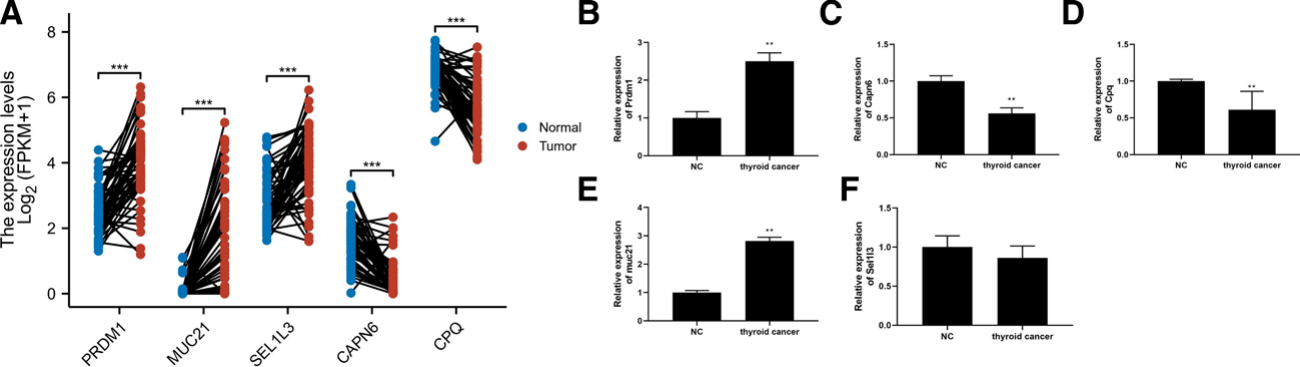
Identification of expression levels of 5-PRs. (A) The differential expression of PRDM1, MUC21, and SEL1L3, CAPN6 and CPQ in TC samples compared to adjacent normal controls in TCGA database. The expression levels of PRDM1 (B), CAPN6 (C), CPQ (D), MUC21 (E), and SEL1L31 (F) 10 pairs of TC and matched adjacent normal tissues were analyzed with qRT-PCR. *P < .05, ***P < .001, ****P < .0001. PRs = phagocytosis related regulators, TC = thyroid cancer.

In order to analyze 5-PRs expression levels in TC samples and paired normal samples, we used TCGA data. According to the results, PRDM1, MUC21, and SEL1L3 expression level were remarkably up regulated in TC samples compared to normal samples. (Fig. 12A). Subsequently, clinical tissue samples were also analyzed using qRT-PCR to detect PRDM1 mRNA levels (Fig. 12B) and MUC21 (Fig. 12E) in TC tissue groups were remarkably increased compared to the normal group, and the CAPN6 mRNA level (Fig. 12C) and CPQ mRNA level (Fig. 12D) in TC tissue were remarkably reduced compared to the normal tissues. Unfortunately, the comparison of expression about SEL1L3 were not significant (Fig. 12F). As a whole, as a result of these outcomes, PRs prognostic signature was further validated as stable and reliable.

## 4. Discussion

The phagocytosis process with target cell identification, cytophagy and lysosomal digestion included involves several steps that are affected by receptor-ligand interactions and the target cell.^[6]^ Though the ability of healthy normal tissues and cells to resist self-elimination by phagocytes has been passed down from generation to generation through the expression of anti-phagocytosis molecules, cancer cells depend even more on similar mechanisms to evade immune eradication.^[24]^ Therefore, for immunotherapy and prognosis prediction of thyroid cancer, PRs must be analyzed comprehensively. Based on CRISPR/CAS9 data, the PRs-signature was used in the research to construct a robust PRs-Score to predict the prognosis of patients with TC. Our bioinformatics study is a follow-up exploration of CRISPR/CAS9 research for robustness, our signature outperformed the current risk scores for TC survival prediction.^[20,21]^ In order to evaluate treatment effectuality, our PRs-signature can evaluate not only the effect of immunotherapy but can also preliminarily explore the possible relationship between specific PRs and ADCP status. Remarkably, when we examined the immune microenvironment, we identified a crucial correlation between PRs risk scores and M2 type macrophages.

In addition, we identified 7 specific PRs with prognostic value, including RDM1, MUC21, SEL1L3, ADD3, CPQ, CAPN6, and CRELD2. PRDM1 is a transcription factor that controls B- and T-cell development and is involved in T-cell-mediated immunosuppression.^[25]^ Growing evidence reveals that PRDM1 expression affects the prognosis of various malignancies in distinct ways, such as multiple myeloma,^[26]^ lung cancer,^[27]^ and glioma,^[28]^ etc. Interestingly, PRDM1 has been revealed to be a novel regulator of macrophage gene expression and function,^[29]^ and can regulate phagocytosis in health or disease.^[30]^ MUC21 was discovered in a human cervical cancer cell line as the human homolog of mice Muc21/epimer.^[31]^ MUC21 influences tumor cell invasiveness and is expressed in a range of malignancies, including lung^[32]^ and esophageal cancers.^[33]^ In addition, SEL1L3 may be related to the prognosis of lung adenocarcinoma,^[34]^ but the underlying molecular mechanisms are not discussed. ADD3 is a critical actin cytoskeleton assembly factor that has been reported to be overexpressed in a variety of malignancies.^[35]^ Although ADD3 has not been explored in depth mechanistically in thyroid cancer or macrophage regulation, studies have positively shown that it can be used as a diagnostic biomarker for papillary thyroid cancer.^[36]^ Meanwhile, CAPN6 is a nonclassical calprotease and is overexpressed in liver cancer^[37]^ and cervical cancer.^[38]^ Many studies have confirmed its important role in multiple biological processes such as proliferation, apoptosis and differentiation.^[39–41]^ Moreover, regarding the CRELD2 gene, only 1 study has demonstrated its role in cancer: the paracrine ROCK-PERK-ATF4-CRELD2 axis promotes the progression of breast cancer and has implications for cancer treatment.^[42]^ Despite the fact that the genes mentioned above play an important role in the progression of cancer, how they affect the development of TC cancer and ADCP is still unclear. Consequently, in vivo and in vitro experiments will be built upon the results of this study.

Nevertheless, as our results have been obtained via bioinformatic analyses, we need further real-world samples for external validation. Additionally, we lack experimental data to confirm the expression, function and mechanism of the specific PRs in TC cell lines. In the future, the development of targeted therapies and the improvement of prognostic prediction can be improved by risk signatures on the basis of PRs altered.

## 5. Conclusion

In order to predict the prognosis of TC patients, we constructed a precise and robust PRs-signature. Using this approach, we discovered specific PRs in TC that interact with each other and macrophage phagocytosis. Besides, the PRs-signature can predict the immunotherapy response and tumor microenvironment. In particular, the molecular subtypes based on NMF algorithm, and there may be a difference in ADCP status among different risk groups in TC patients. In short, this will contribute to the development of new therapeutic strategies and lead to better survival rates for patients with TC.

## Author contributions

**Data curation:** Changran Hou.

**Funding acquisition:** Zhenlin Yang.

**Investigation:** Mengmeng Wu.

**Methodology:** Mengmeng Wu.

**Software:** Zhenlin Yang.

**Supervision:** Haojie Zhang.

**Writing – original draft:** Changran Hou.

**Writing – review & editing:** Zhenlin Yang.
